# Melatonin Promotes SGT1-Involved Signals to Ameliorate Drought Stress Adaption in Rice

**DOI:** 10.3390/ijms23020599

**Published:** 2022-01-06

**Authors:** Ruiqing Li, Ruifang Yang, Wenyin Zheng, Liquan Wu, Can Zhang, Huali Zhang

**Affiliations:** 1 College of Agronomy, Anhui Agricultural University, Hefei 230036, China; liruiqing@ahau.edu.cn (R.L.); zhengwenyin_75@163.com (W.Z.); wuliquan@ahau.edu.cn (L.W.); 18656159363@163.com (C.Z.); 2 State Key Laboratory of Rice Biology, Chinese National Center for Rice Improvement, China National Rice Research Institute, Hangzhou 310029, China; 3 Crop Breeding and Cultivation Research Institute, Shanghai Academy of Agricultural Sciences, Shanghai 201403, China; yangruifang1982@163.com

**Keywords:** *Oryza sativa* L., drought stress, melatonin, SGT1, ABI5, signals

## Abstract

Drought has become one of the environmental threats to agriculture and food security. Applications of melatonin (MT) serve as an effective way to alleviate drought stress, but the underlying mechanism remains poorly understood. Here, we found that foliar spray of 100-µM MT greatly mitigated the severe drought stress-induced damages in rice seedlings, including improved survival rates, enhanced antioxidant system, and adjusted osmotic balance. However, mutation of the *suppressor of the G2 allele of skp1* (*OsSGT1*) and *ABSCISIC ACID INSENSITIVE 5* (*OsABI5*) abolished the effects of MT. Furthermore, the upregulated expression of *OsABI5* was detected in wild type (WT) under drought stress, irrespective of MT treatment, whereas *OsABI5* was significantly downregulated in *sgt1* and *sgt1abi5* mutants. In contrast, no change of the *OsSGT1* expression level was detected in *abi5*. Moreover, mutation of *OsSGT1* and *OsABI5* significantly suppressed the expression of genes associated with the antioxidant system. These results suggested that the functions of *OsSGT1* in the MT-mediated alleviation of drought stress were associated with the ABI5-mediated signals. Collectively, we demonstrated that OsSGT1 was involved in the drought response of rice and that melatonin promoted SGT1-involved signals to ameliorate drought stress adaption.

## 1. Introduction

Plants being sessile are particularly susceptible to various stressful environments, including abiotic stress (i.e., drought, chilling, heavy metal, etc.) and biotic stress (i.e., insect and disease damages) [1,2]. The supply of adequate soil moisture is essential for plant growth, while water shortage greatly restrict the plant developmental process [3,4]. In recent years, with the frequent climate extremes, drought stress episodes have intensified crop production worldwide, especially in drought-prone arid and semiarid areas [5]. Therefore, drought is becoming a daunting challenge for global agriculture [6]. For example, it has been announced that China is now a drought-effect country, with a 14.7% drought-induced economic loss of the total GDP in the past decade [7]. Thus, drought has become one of the environmental threats to agriculture and food security.

Drought stress can induce the hyperaccumulation of oxidant products [8,9], i.e., reactive oxygen species (ROS), hydrogen peroxide (H_2_O_2_), malondialdehyde (MDA), and superoxide (O_2_^−^), leading to oxidative damages to cell constitutes [10]. Plants have evolved a set of antioxidant systems to protect plants from drought damages [11], including enzymatic ones, i.e., superoxide dismutase (SOD, EC 1.15.1.1), catalase (CAT, EC 1.11.1.6), ascorbate peroxidase (APX, EC 1.11.1.11), and glutathione reductase (GR, EC 1.6.4.2), and nonenzymatic molecules, i.e., glutathione (GSH) and ascorbic acid (ASA). Moreover, some osmolytes, i.e., soluble sugar and proline, are also triggered to adjust leaf moisture levels through stomatal movement under drought stress [12].

Various strategies aimed to enhance the antioxidant system serve as effective ways to combat drought stress. Although many abiotic stress-associated genes have been identified in plants, the efforts to improve drought tolerance have mostly failed by using genetic engineering strategy [2], which is also probably limited by the current national legislation. Nonetheless, exogenous applications of various chemicals are also effective and commonly used ways to ameliorate adaptions to drought stress, such as nanoparticles [13], abscisic acid (ABA) [14], glutathione [15], melatonin (MT) [6,16], and free proline [17]. Until now, the alleviated effects of melatonin on drought adaption have been well-documented in many plants. Thus, melatonin has recently attracted more attention and is thought to be a potential multifunctional biomolecule for tolerant drought stress [6].

The studies conducted so far on the potential of melatonin to alleviate drought stress mainly focused on the improved photosystem capacity [9] and the enhanced antioxidants for ROS scavenging in various species [16]. For example, enhanced efficiency of photosystem II and increased photosynthesis after foliar spraying with MT have been found in several cereal crops, such as wheat [9], soybean [18,19], and maize [20]. Besides, reduced ROS accumulation via a regulated antioxidant system serves as another key indicator to explain the mitigating effects of melatonin on drought stress [16,21]. Aside from these effects, phytohormone-mediated signals were also usually thought to be responsible for the melatonin-alleviated effects on drought stress. Melatonin cross talks with other plant hormones, i.e., indole-3-acetic acid (IAA), ABA, gibberellins (GA), jasmonic acid (JA), and salicylic acid (SA), to drive system-acquired response to various stresses [22,23,24]. Among them, ABA has been widely reported to serve as one vital signalling molecule under drouth stress conditions in many plants, whose effect is largely associated with the enhanced antioxidant system [14,25]. Interestingly, melatonin antagonistically interacts with ABA to alleviate drought stress [26,27] while promoting the synthesis of other hormones, i.e., SA, JA, even under drought stress [6,19].

Despite these findings, the mechanisms underlying the alleviating effects of melatonin on drought stress in rice remains largely unknown. Rice is one of the most important staple food crops to feed the global population, especially in Asian countries [28]. Because of its semiaquatic patterns, rice growth requires a relatively high soil moisture level [29], while water deficiency causes depressed seedling growth and reduced rice productivity [3,4]. However, due to the varying water availability in the planting environments, it is still a breeding challenge to improve the drought tolerance of rice cultivars via breeding strategies [28,30] or applications of exogenous compounds, i.e., glutathione [15], proline [17], and others. Nonetheless, there are limited reports on the melatonin-induced adaption to drought stress in rice. Interestingly, *SGT1* (termed *suppressor of the G2 allele of skp1*) plays vital roles in plant disease resistance (R) [31,32], salt tolerance [33,34], and heat tolerance [35,36]. We found that mutation of *OsSGT1*, one homologous cochaperone of *SGT1*, caused ROS accumulation and negatively affected the photosynthetic capacity of rice seedlings under drought stress, even with melatonin treatments. Therefore, we further aim to examine how melatonin ameliorates drought adaption through an SGT1-mediated signal, which would provide crucial guidance in applying melatonin in rice production.

## 2. Results

### 2.1. Determination of Optimal Concentration of Melatonin on Drought Stress

To investigate the effects of drought stress on the survival rates of seedlings, various degrees of drought stress conditions, including 100% field capacity (FC; control), 80% FC (slight), 60% FC (mild), 40% FC (middle), and 25% FC (severe), were employed to treat seedlings of wild-type (WT). Within the investigated time, compared with the control (CK), all levels of drought stress negatively affected the survival rates of seedlings. Compared with control, the survival rates were greatly reduced by 11.99%, 46.11% and 75.51% under 80% FC, 60% FC, 40% FC conditions, respectively (Figure S1). Nonetheless, there was little difference in survival rates between control and the slight conditions of 80% FC, while the severe drought stress of 25% FC caused 74.57–90.03% decreases in survival rates, compared to CK (Figure S1).

We next select 25% FC to assess the optimal concentration of melatonin needed to improve drought tolerance. Various concentrations of melatonin, including 50-, 100-, 200-, and 1000-µM melatonin, were used in foliar spraying applications on seedlings under drought stress. Compared with the single drought-stressed ones, all the employed melatonin concentrations showed improvements in seedlings’ survival rates (Figure S2). Among them, applications of 50-µM melatonin had slight effects on the survival rates, while high concentrations of 1000-µM melatonin had minimal, even lower than with 50-µM (Figure S2). In contrast, both 100- and 200-µM melatonin significantly increased seedling survival rates, and no appreciable difference in those rates were found between them (Figure S2). Since 25% FC had the most severe effects on survival rates of seedlings, and the application of 100-µM melatonin was effective in countering the effects of drought stress on the survival rates of seedlings, the 100-µM melatonin concentration was eventually used as the optimal concentration to ameliorate severe drought stress (drought).

### 2.2. Effects of Melatonin on the Seedling Performance in sgt1 Mutants under Drought Stress

**Figure 1:**
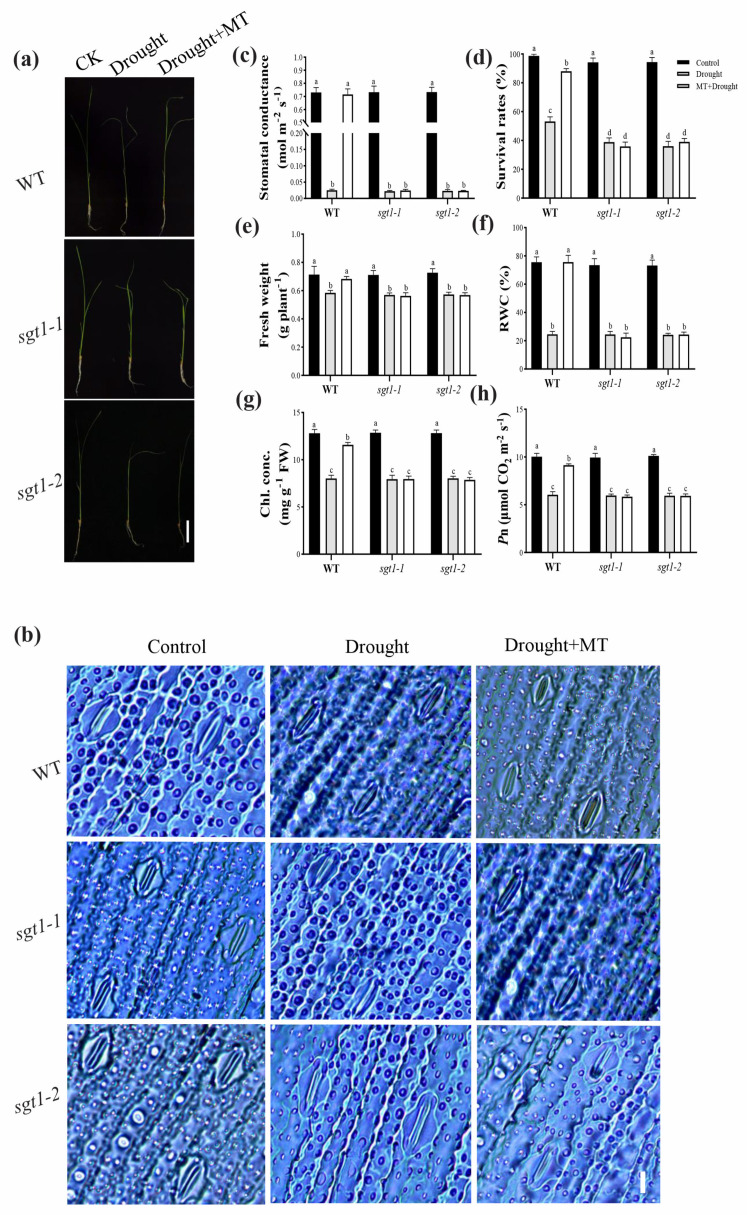
Effects of melatonin treatments on seedling performance under drought stress. (**a**) Plant phenotype, (**b**) stoma phenotype, (**c**) stomatal conductance, (**d**) survival rate, (**e**) fresh weight, (**f**) relative water contents (RWC), (**g**) chlorophyll (Chl) contents, and (**h**) photosynthetic rates (*P*_n_) were examined in the leaves of wild-type (WT) and *sgt1* mutant seedlings treated with melatonin (MT; 100 μM) under drought stress. Difference in letters indicates significant differences according to Tukey’s Multiple Comparison Test (*p* < 0.05). (**a**) Bar = 3 cM; (**b**) Bar = 10 μM.

Drought stress usually inhibits seedling growth and disrupts photosynthesis and physiological metabolism. Melatonin has been reported to mitigate the adverse effects of drought stress on seedlings [6,16]. To determine whether the *OsSGT1* mutation affected the mitigating effects of melatonin on seedlings’ growth under drought stress, plant phenotypes, stomatal aperture, survival rates, plants height, fresh weight, relative water contents (RWC), chlorophyll (Chl.) contents and photosynthetic rates (*P*_n_) were investigated in WT and *sgt1* mutants. Drought stress conditions led to the inhibited seedlings growth (Figure 1a), closure of stoma (Figure 1b), and lower stomatal conductance (Figure 1c), and also significantly decreased the entire investigated index (Figure 1d–h; Figure S3), both in WT and *sgt1*. Significantly, compared with the control, the survival rates were decreased by 46.12% and 59.3% in WT and *sgt1*, respectively (Figure 1d), while RWC were reduced by 67.28% and 66.76% in WT and *sgt1*, respectively (Figure 1f). Moreover, the contents of chlorophylls and the photosynthetic rates under drought stress were significantly lower than control (Figure 1g,h). These results suggested that the seedling growth was greatly suppressed under drought stress.

Notably, after applications of foliar spraying with MT, the seedings’ growth in WT was significantly restored, though still weaker than CK, and stoma remained opening (Figure 1a–c,e; Figure S3). All other investigated physiological attributes of WT were also significantly increased in the presence of MT treatment, compared to untreated ones (Figure 1d,f–h). In contrast, under drought stress, no significant difference was detected in the seedling performance in *sgt1* mutants, irrespective of MT feeding, suggesting that the *OsSGT1* mutation greatly affected the alleviating effects of melatonin on drought-stressed seedling growth (Figure 1d,f–h).

In addition, drought stress caused the increased contents of free proline and soluble sugar (SS) in WT, indicating that drought stress led to osmotic stress, and compared with the control, the melatonin treatment significantly suppressed their contents (Figure S4). However, under drought conditions, proline and soluble sugar contents in *sgt1* remained at higher levels than in CK, irrespective of the MT treatment (Figure S4). These results suggested that under drought stress, the *OsSGT1* mutation led to an accumulation of proline and soluble sugar, even in the conditions of melatonin treatment, which would cause osmotic stress.

### 2.3. Effects of Melatonin on the Oxidative Damages in sgt1 Mutants under Drought Stress

**Figure 2:**
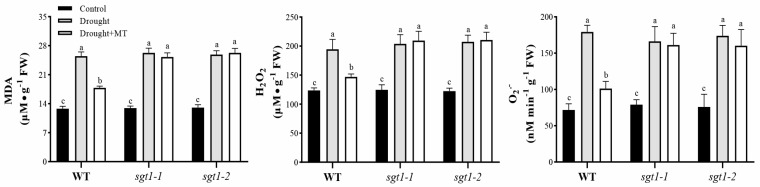
Effects of melatonin treatments on oxidative damages under drought stress. Contents of hydrogen peroxide (H_2_O_2_) and malondialdehyde (MDA), and superoxide (O_2_^−^) production rates, were examined in the leaves of wild-type (WT) and *sgt1* mutant seedlings treated with melatonin (MT; 100 μM) under drought stress. Difference in letters indicates significant differences according to Tukey’s Multiple Comparison Test (*p* < 0.05).

Osmotic stress caused by drought stress disrupts the redox homeostasis, which eventually causes the accumulation of oxidant products. Melatonin has been widely reported to suppress the drought stress-induced production of ROS [10]. Thus, to detect the effects of *OsSGT1* mutation on the melatonin mitigated effects under drought stress, contents of the oxidant products, including MDA, H_2_O_2_, and O_2_^−^, were measured in WT and *sgt1*. Under nonstressed conditions, no significant difference was detected in the content of oxidant products between WT and *sgt1* (Figure 2). However, under drought stress conditions, compared with the control, the contents of MDA and H_2_O_2_ and the O_2_^−^ production rates were significantly increased in both WT and *sgt1* (Figure 2). After the MT treatment, all the investigated index was significantly decreased under drought stress in WT, though still higher than CK (Figure 2). However, under drought stress conditions, the content of MDA and H_2_O_2_, and the O_2_^−^ production rates, there was no significant change in *sgt1* mutants, regardless of whether melatonin treatment was administered (Figure 2). These results indicate that melatonin can suppress the drought stress-induced production of oxidant products, which failed in the absence of *OsSGT1*.

### 2.4. Effects of Melatonin on the Antioxidant Activity under Drought Stress

**Figure 3:**
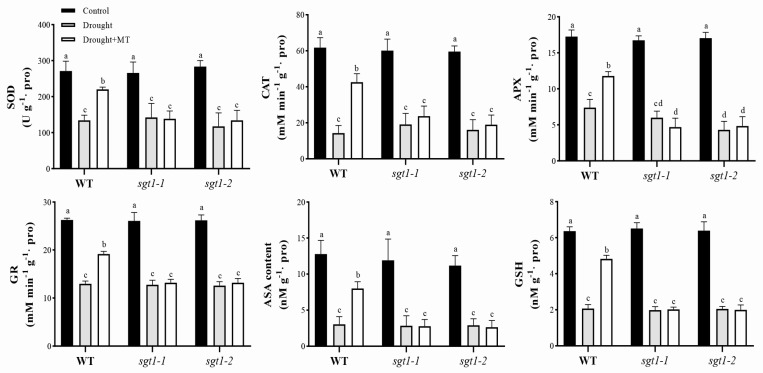
Effects of melatonin treatments on the antioxidant activity under drought stress. Activities of superoxide dismutase (SOD), catalase (CAT), ascorbate (APX), and glutathione reductase (GR), as well as the ascorbic acid (ASA) and glutathione (GSH) contents, were examined in the leaves of wild-type (WT) and *sgt1* mutant seedlings treated with melatonin (MT; 100 μM) under drought stress. Difference in letters indicates significant differences according to Tukey’s Multiple Comparison Test (*p* < 0.05).

Oxidative products, i.e., H_2_O_2_, could activate the antioxidant system to maintain redox homeostasis, but excess accumulation would damage the antioxidant system [37]. Melatonin is thought to promote the antioxidant system to scavenge ROS, even under severe drought stress, such as 25% FC [38]. To detect whether the mutation of *OsSGT1* affected the promoted effects of melatonin on antioxidant system under drought stress, activities of the enzymatic SOD, CAT, APX, GR, and content of the nonenzymatic ASA and GSH, were further investigated. There was no significant difference in the antioxidant systems detected between WT and *sgt1* mutants under unstressed conditions (Figure 3). After being transferred to conditions of drought stress, activities of SOD, CAT, APX, GR in WT were greatly decreased, compared with CK, whereas in the presence of MT treatment, their activities were restored by 68.34–81.14%, a significant improvement similar to CK levels, though still lower (Figure 3). In contrast, no significant difference was found in *sgt1-1* and *sgt1-2* mutants under drought stress, irrespective of the MT treatment, displaying weaker activities than CK (Figure 3).

Accordingly, similar trends were seen in the contents of ASA and GSH. Under drought stress, contents of ASA and GSH were significantly decreased in WT seedlings but greatly restored, up to 62.68% and 75.79% compared with CK, after the MT treatment (Figure 3). In contrast, although decreased contents of ASA and GSH were detected in *sgt1* mutants under drought stress, the treatment of melatonin had no significant effect on them (Figure 3). These results indicated that melatonin can greatly alleviate the damages of drought stress to the antioxidant system but can be significantly affected by the mutation of *OsSGT1*.

### 2.5. Effects of Melatonin on the Synthesis of Endogenous ABA, SA and JA under Drought Stress

**Figure 4:**
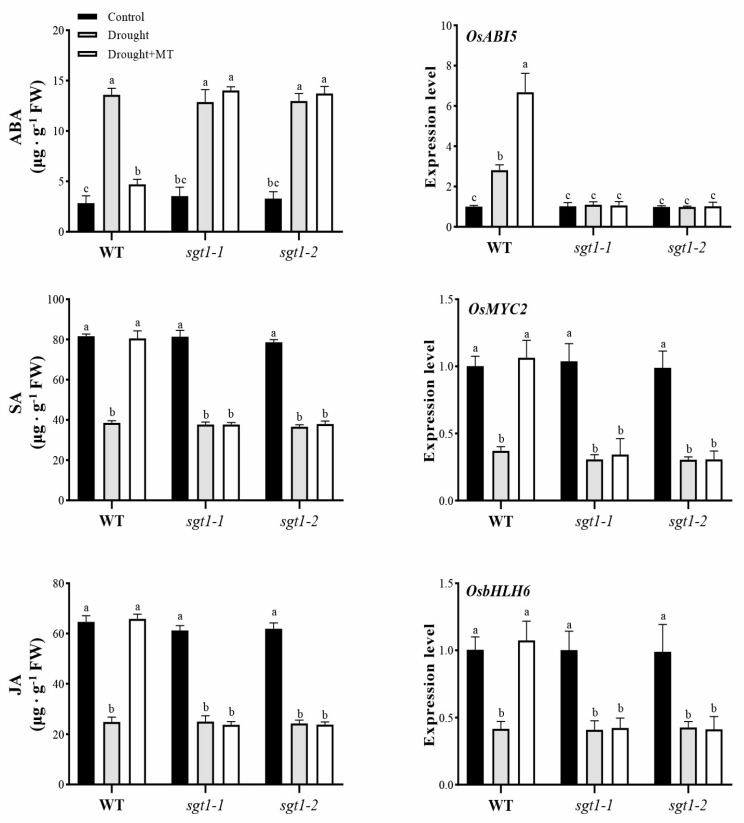
Effects of melatonin treatments on the endogenous phytohormones biosynthesis under drought stress. Contents of abscisic acid (ABA), salicylic acid (SA) and jasmonic acid (JA), as well as expression levels of *OsABI5*, *OsMYC2* and *OsbHLH6,* were examined in the leaves of wild-type (WT) and *sgt1* mutant seedlings treated with melatonin (MT; 100 μM) under drought stress. Difference in letters indicates significant differences according to Tukey’s Multiple Comparison Test (*p* < 0.05).

Melatonin has been well-documented to tolerate drought stress via the crosstalk with other phytohormones, i.e., ABA, SA, and JA [37]. To further detect the effects of *OsSGT1* mutation on the crosstalk of MT with other phytohormones under drought stress, contents of ABA, JA, and SA were investigated. Under optimal conditions, no significant differences in the three investigated phytohormones were found between WT and *sgt1* mutants (Figure 4). However, compared with CK, the ABA content was significantly increased under drought stress, by 4.79 folds and 3.29–3.94 folds in WT and *sgt1*, respectively, whereas the contents of SA and JA were significantly decreased in both WT and *sgt1* mutant (Figure 4). In contrast, the MT treatment greatly restored the contents of ABA, SA, and JA, almost to the levels of CK, in WT but had no effect in *sgt1-1* and *sgt1-2* mutants (Figure 4). This suggests that the mutation of *OsSGT1* affected MT-induced synthesis of endogenous ABA, JA, and SA under drought stress.

Expressions of ABA-, JA- and SA-associated signalling genes were also investigated, including *abscisic acid insensitive 5* (*OsABI5*), *basic helix-Loop-helix protein 6* (*OsbHLH6*), and *myelocytomatosis oncogene transcription factor 2* (*OsMYC2*). Consequently, upregulated *OsABI5* expression and downregulated expression of *OsbHLH6* and *OsMYC2* were detected in WT under drought stress. The treatment of MT led to a restored expression of all three investigated genes even under conditions of drought stress (Figure 4). Specifically, MT-induced *OsABI5* expression was even higher than under-drought stress, where no significant difference in the expression of *OsABI5*, *OsMYC2*, and *OsbHLH6* was detected in *sgt1-1* and *sgt1-2* mutants, irrespective of MT treatment (Figure 4). All these results indicated that the mutation of *OsSGT1* had significant effects on the interrelationship of MT with other phytohormones under drought stress.

### 2.6. Effects of Dual Mutation of OsABI5 and OsSGT1 on the Melatonin-Alleviated Drought Stress

**Figure 5:**
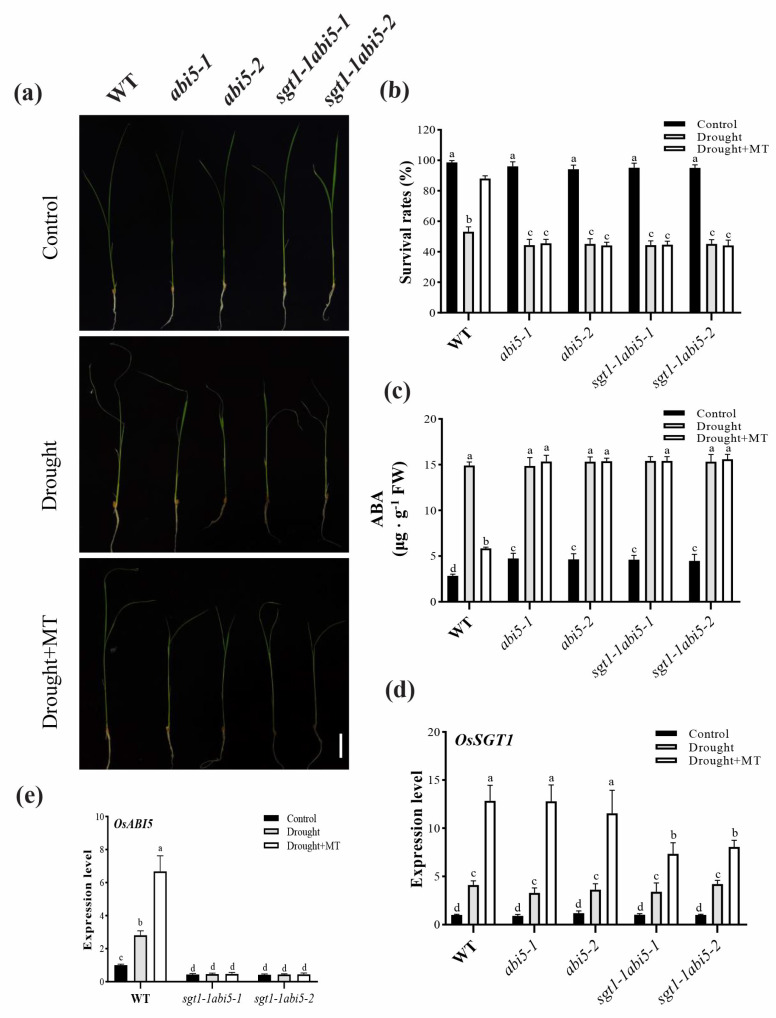
Effects of dual mutation of *OsSGT1* and *OsABI5* on the melatonin-alleviated drought stress. (**a**) Plant phenotype, (**b**) survival rates, (**c**) ABA contents, and the expression levels of (**d**) *OsSGT1* and (**e**) *OsABI5*, were examined in the leaves of wild-type (WT), *sgt1, abi5*, and *sgt1-1abi5* mutant seedlings treated with melatonin (MT; 100 μM) under drought stress. Difference in letters indicates significant differences according to Tukey’s Multiple Comparison Test (*p* < 0.05). Bar = 3 cM.

ABI5 serves as ABA-mediated signalling under various environmental stresses [37,39]. The above results showed that drought stress led to increased ABA content and upregulated expression of *OsABI5*. Following the treatment with melatonin, the decreased contents of ABA was not consistent with the higher expression of *OsABI5*, which was abolished by the mutation of *OsSGT1* (Figure 4). Therefore, to clarify the relationship between *OsSGT1* and *OsABI5* under drought stress, we investigated the seedlings’ performance in *abi5* and *sgt1-1abi5* mutants. The seedlings of *abi5* and *sgt1-1abi5* showed weak growth and decreased survival rates under drought stress, irrespective of melatonin treatment (Figure 5a,b). In addition, compared with CK, the ABA contents were significantly increased in *abi5* and *sgt1-1abi5* mutants under drought stress, but no significant difference was detected after melatonin treatment (Figure 5c).

Moreover, under drought stress, *OsSGT1* showed higher expression levels than CK and even reached the highest expression levels after MT treatment, despite showing no significant difference in WT, *abi5*, and *sgt1-1abi5* seedlings (Figure 5d). However, in *sgt1-1abi5-1* and *sgt1-1abi5-2* mutants, there was no significant change in *OsABI5* expression levels detected under drought stress, even after melatonin treatments (Figure 5e). These results suggested that mutations of *OsABI5* and *OsSGT1* affected the alleviating effects of melatonin on drought stress.

Oxidant products and antioxidant systems were further investigated in *abi5* and *sgt1-1abi5* to detect the relationship between OsSGT1 and OsABI5 during drought stress. Compared with CK, contents of MDA/H_2_O_2_ were increased by 2.75–2.81/1.94–1.95-folds and 2.48–2.82/1.92–1.97-folds in *abi5* and *sgt1-1abi5*, respectively (Figure S5). Similarly, the production rates of O_2_^−^ was also greatly induced in *abi5* and *sgt1-1abi5* under drought stress compared to CK (Figure S5). Nonetheless, no significant difference in the investigated oxidant products was detected after melatonin treatments in both of *abi5* and *sgt1-1abi5* mutants (Figure S5). Activities of the antioxidant system showed antagonistic dynamic changes in the performance of oxidant products (Figure S6). Although no difference in the antioxidant system was detected between WT and the mutants of *abi5* and *sgt1-1abi5* under unstressed conditions, activities of SOD, CAT, APX, GR, as well as the contents of GSH and ASA, were significantly decreased in *abi5* and *sgt1-1abi5* mutants under drought stress, even in the melatonin-treated ones (Figure S6). These results indicated that mutation of *OsABI5* and *OsSGT1* affected the alleviated effects of melatonin on drought stress via the disrupted antioxidant system.

### 2.7. Effects of Exogenous ABA Feeding on the Expression Levels of OsABI5 and OsSGT1 under Drought Stress

**Figure 6:**
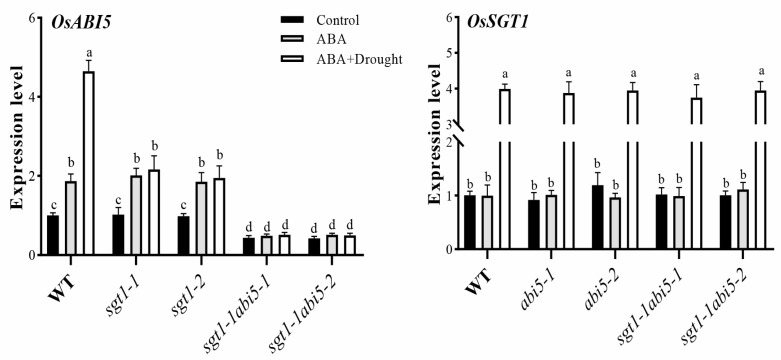
Effects of exogenous ABA on the expression levels of *OsABI5* and *OsSGT1*. Expression levels of *OsABI5* and *OsSGT1* were examined in the leaves of wild-type (WT), *abi5*, *sgt1-1abi5* and *sgt1* mutant seedlings treated with exogenous ABA (10 μM) under drought stress. Difference in letters indicates significant differences according to Tukey’s Multiple Comparison Test (*p* < 0.05).

To further detect the effects of ABA on the OsSGT1-involved drought adaption, expression of *OsSGT1* and *OsABI5* were investigated after feeding with exogenous ABA in *sgt1*, *abi5* and *sgt1-1abi5* mutants. Compared with CK, expression of *OsABI5* was significantly induced by exogenous feeding of ABA and remained consistent expression or became even much higher expression in WT and *sgt1* after drought stress (Figure 6). By contrast, in dual mutants of *sgt1-1abi5-1* and *sgt1-1abi5-2*, the expression of *OsABI5* showed no significantly different expression, irrespective of ABA feeding and drought stress treatments (Figure 6). However, there was no significant difference in the *OsSGT1* expression levels found in all investigated seedlings under non-stressed conditions, no matter ABA treatment or not, though the higher expression of *OsSGT1* under drought stress were found in WT, *abi5* and *sgt1-1abi5* as compared to CK (Figure 6). Thus, the ABA feeding greatly promoted the expression of *OsABI5* but had no effects on *OsSGT1*, while the mutation of *OsSGT1* largely affected the *OsABI5* expression, indicating that melatonin promoted *OsSGT1* signals to induce the expression of *OsABI5* via an ABA-independent way under drought stress.

### 2.8. Effects of Dual Mutation of OsABI5 and OsSGT1 on the Melatonin-Induced Expression of Antioxidant Genes under Drought Stress

**Figure 7:**
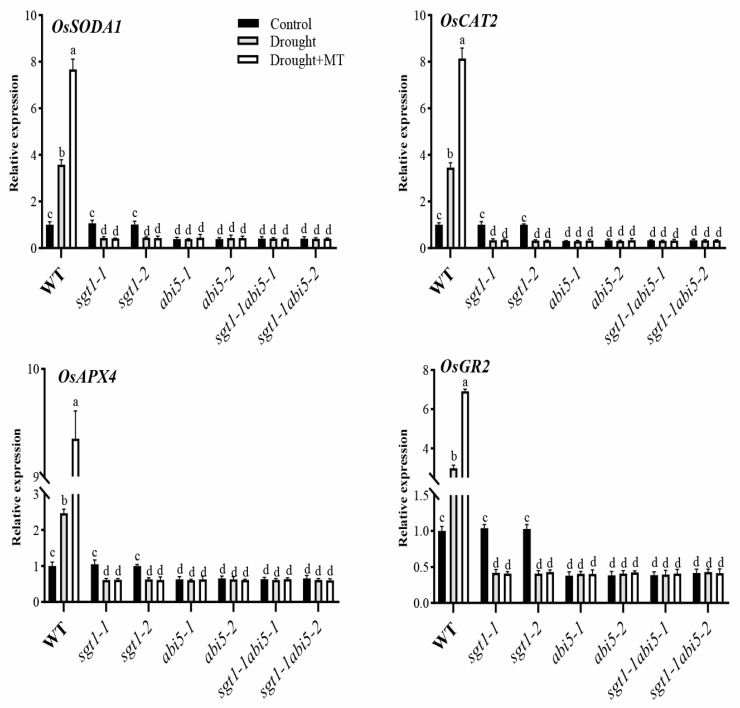
Effects of melatonin treatments on the expression level of antioxidative genes under drought stress. Expression of *superoxide dismutase A1* (*OsSODA1*), *catalase 2 (OsCAT2), peroxisomal ascorbate peroxidase 4* (*OsAPX4*), and *glutathione reductase 2* (*OsGR2*) were examined in the leaves of wild-type (WT), *sgt1*, *abi5* and *sgt1-1abi5* mutants treated with melatonin (MT; 100 μM) under drought stress. Difference in letters indicates significant differences according to Tukey’s Multiple Comparison Test (*p* < 0.05).

Expression of antioxidant responsive genes, including *superoxide dismutase A1* (*OsSODA1*), *peroxisomal ascorbate peroxidase 4* (*OsAPX4*), *glutathione reductase 2* (*OsGR2*), and *catalase 2 (OsCAT2)*, were further investigated to confirm the effects of *OsSGT1* and *OsABI5* on the antioxidant system. Compared with CK, in WT, expressions of the four investigated genes were significantly upregulated under drought stress while becoming even higher after melatonin treatment (Figure 7). In sharp contrast to WT, drought stress greatly down-regulated the expression of *OsSODA1*, *OsCAT2*, *OsAPX4* and *OsGR2* in *sgt1-1* and *sgt1-2* mutants, irrespective of MT treatment (Figure 7). Moreover, in *abi5* mutants, as wells as the dual mutants of *sgt1-1abi5*, there was no significant difference in the expression level of these genes, irrespective of the drought stress and melatonin treatment (Figure 7). These results indicated that the effects of *OsSGT1* and *OsABI5* on the antioxidant system were tightly associated with the expression of antioxidant responsive genes, i.e., *OsSODA1*, *OsCAT2*, *OsAPX4* and *OsGR2***.**

## 3. Discussion

### 3.1. Melatonin Alleviates Drought Stress to Promote Seedling Growth

Drought stress damages seedlings’ growth through the over-excessed accumulation of ROS [8,10], which can also lead to the disruption of the antioxidant system [16] and photosynthetic capacity [9]. Applications of MT can effectively alleviate the drought damages in many agricultural crops, such as wheat [9], maize [20], soybean [18,19], rapeseed [21], and cotton [40]. However, limited reports have been shown in rice. We here reported that drought stress induced the retarded growth of seedlings or even lethal phenotypes, whereas foliar spray applications of 100-µM MT greatly mitigated the severe drought stress-induced damages to rice seedlings (Figure 1, Figures S1–S4). Consistent with previous reports, the alleviated effects of melatonin on drought stress in this study were mainly focused on the following aspects, including (i) the improved capacity of the photosynthetic system (Figure 1g,h), (ii) the restored stomatal phenotype and conductance (Figure 1b,c) and water utilization (Figure 1f), (iii) the enhanced antioxidant system (Figure 3), and (iv) the adjusted osmotic substances, i.e., ABA, SS and free proline (Figure 4 and Figure 5; Figure S4). Inhibited growth of the plant was largely attributed to the essential role of water in cell turgor [2]. Melatonin can enhance the stomatal conductance to improve the availability of water to ameliorate drought adaption [41,42]. We also found that melatonin significantly increased the RWC of seedlings (Figure 1f) and maintained the stomatal conductance (Figure 1b,c), suggesting that the alleviated effects of melatonin on drought stress was associated with stoma movement.

Accumulation of osmolytes, such as SS and proline, was expected to adjust osmotic stress during drought stress [12]. Subsequently, some studies also reported that enhanced soluble sugars were detected after the treatment of melatonin [43]. Others demonstrated that accumulated osmolytes caused osmotic stress under abiotic stress, which was related to the redox homeostasis by exogenous melatonin [44,45]. The current results were aligned with the latter studies, showing decreased accumulation of SS and proline after the MT treatment under severe drought stress (Figure S4). Lei et al. [44] proposed that such MT-alleviated effects were not associated with osmotic adjustment. Besides, another possible explanation was that the disruption of ROS homeostasis under severe drought stress caused depressed functioning of cell metabolism.

The enhanced effects of melatonin on drought adaption have been well-documented via the enhanced antioxidant system in many plants [6]. The present study also found that foliar spray of melatonin to rice leaves greatly improved the activities of the antioxidant system (Figure 3) and vastly decreased the contents of oxidant products (Figure 2). Aside from enzymatic antioxidants, i.e., SOD, APX, CAT, and GR, the nonenzymatic ASA–GSH biosynthesis was also significantly increased after the melatonin treatment (Figure 3). ASA–GSH is associated with abiotic tolerance in higher plants [46], and the improved tolerance of wheat seedlings to drought stress has been reported to largely depend on the regulated ASA–GSH biosynthesis [14]. Our study validated these findings (Figure 1 and Figure 3), and we confirm that melatonin can mitigate the damages of drought stress to rice seedlings.

### 3.2. SGT1 Is Crucial for the Melatonin Alleviated Effects on Drought Stress

SGT1, firstly identified in yeast, is a suppressor of the G2 allele of skp1 and is highly conserved in plants and mammals [32]. It is involved in thermotolerance and has been shown to function in plant disease resistance (R) [31] and in response to various abiotic stresses, such as salt [33], heat [34,35,36], and drought [34]. *BolSGT1* showed enhanced expression levels under drought treatment in *Brassica oleracea* [34]. We also found that the expression level of *OsSGT1* was sensitive to drought stress in rice seedlings (Figure 5d and Figure 6). Although the applications of melatonin alleviated the effects of drought-induced damages to seedlings, the mutation of *OsSGT1* greatly affected these melatonin-mitigated effects, leading to inhibition of the antioxidant system and accumulation of oxidant products (Figure 1, Figure 2 and Figure 3) and suggests its important roles under drought stress.

Moreover, following previous reports, our results suggest that OsSGT1 possibly serves as one module in melatonin-mediated signals and that melatonin responds to drought stress through the regulation of *OsSGT1* because the *OsSGT1* mutation essentially abolished the alleviating effects of melatonin, resulting in retarded growth, accumulation of ROS, etc. (Figure 1 and Figure 2). Another possible explanation was that melatonin had an effect on the expression level of *OsSGT1* via affecting other phytohormones, such as ABA. However, accumulated contents of ABA did not alter the expression of *OsSGT1* in *abi5 and sgt1–1abi5* mutants and had no effect on *OsSGT1* expression levels (Figure 5). This was also confirmed in the exogenous feeding of ABA treatments, where ABA produced no significant difference in *OsSGT1* expression (Figure 6). Previous reports also demonstrated that the expression of *OsSGT1* was not induced by ABA but by JA or SA [47]. As the plant signalling molecules, JA and SA have been well documented to play vital roles in response to various stresses, including drought [19]. Moreover, JA has been reported to control water loss via stomata adjustment [12], while SA also serves as a receptor to provide SGT1-involved signalling in *Arabidopsis* [32]. In the current study, SA, JA, and their mediated signals were significantly suppressed in *sgt1* mutants, even after the melatonin treatments (Figure 4), suggesting the tight association of OsSGT1 with SA and JA. This study extends the roles of OsSGT1 and shows that SGT1 is vital to the melatonin-alleviated effects under drought stress.

### 3.3. The Ameliorated Effects of Melatonin on Drought Adaption Is Tightly Associated with SGT1-Involved Signals

Although it is widely recognized that melatonin mitigates drought stress in many plants [6], its molecular mechanism is still poorly understood. Nonetheless, some mechanisms have been proposed to explain the functions of melatonin under various abiotic stress. Hasan et al. [48] demonstrated that melatonin employed an enhanced antioxidant system and regulated sulfur metabolism to scavenge ROS and eventually enhance cadmium tolerance in tomato seedlings. Li et al. [49] reported that the alleviated effects of melatonin under low-temperature stress was primarily associated with ABI5-mediated signals, which further activate the antioxidant system. Recently, melatonin has been reported to antagonize with ABA to promote seed germination via Ca^2+^ efflux [50]. Therefore, since the antioxidant system usually acts as the scavengers of ROS under various stresses [6], melatonin-mediated tolerance to stress greatly depends on redox homeostasis.

Although significant levels of ROS leading to mild oxidative stress is beneficial to stress tolerance, excessive ROS would damage cellular components and affect plant development through the alternations of cellular redox homeostasis [51]. SGT1 is involved in the tolerance to various stress, including drought [34], but whether its function in drought stress is ROS-dependent has not yet been revealed in rice. We found that *OsSGT1* was greatly induced under drought stress, even after melatonin treatment (Figure 5). Thus, we proposed that SGT1 is involved in regulating antioxidant system-associated genes (ASAGs) to realize MT-enhanced tolerance to drought stress. This was confirmed with the results from the mutation of *OsSGT1*. Mutation of *OsSGT1* significantly repressed the antioxidant system (Figure 3) via downregulated expression of ASAGs (Figure 7). However, as a chaperone protein [32], SGT1 cannot directly bind to the promoters to regulate gene expression.

Previous reports showed that melatonin mitigated abiotic stress through ABI5-regulated expression of antioxidant genes, i.e., *OsCAT2*, which encodes the CAT enzyme [49]. Interestingly, in the current study, the mutation of *OsSGT1* greatly depressed the expression of *OsABI5*, but not in versus (Figure 5 and Figure 6), suggesting that roles of OsSGT1 in drought stress may be realized through *OsABI5*. Moreover, dual mutation of *OsSGT1* and *OsABI5* also indicated elimination of signals of melatonin-alleviated tolerance to drought (Figure 5, Figure 6 and Figure 7), the disruption of the antioxidant system (Figure S6), and accumulation of oxidative products, i.e., MDA and H_2_O_2_ (Figure S5). ABI5 is one of the downstream signal components of ABA, while its mutation causes insensitive phenotypes to ABA [31,39]. We found that both *abi5* and *sgt1*-1*abi5* mutants showed no response to the accumulated endogenous ABA (Figure 5) and exogenous feeding of ABA (Figure 6), irrespective of drought stress. More importantly, the response of the antioxidant system (Figure 3 and Figure S6) and expression of antioxidant genes (Figure 7) were prone to be fully or partially lost in either *sgt1* or *abi5* mutants under drought stress, even in the conditions of melatonin treatments. In contrast, after melatonin treatment, drought-induced *OsABI5* and *OsSGT1* showed ever higher expression levels in WT, despite the reduced ABA contents (Figure 5), indicating the adjusted functions of melatonin on *OsABI5* and *OsSGT1*.

Crosstalk between MT and other phytohormones also demonstrated this point. Under drought stress, feeding melatonin greatly promoted the synthesis of SA and JA but suppressed the ABA synthesis (Figure 4), which is consistent with the previous reports [19,47,52]. Moreover, expression of *SGT1* has been reported to be induced by JA, SA, and drought stress [19,30,47,52], while *ABI5* was induced by ABA [53,54], as well as exogenous melatonin [49]. We here also found that the expression of *OsSGT1* and *OsABI5* were both induced by melatonin under drought stress (Figure 5 and Figure 6), which was consistent with the synthesis of SA, JA, and ABA (Figure 4). Thus, melatonin promoted the expression of *OsSGT1* and *OsABI5* to activate the antioxidant response; simultaneously, although melatonin inhibited the synthesis of drought-induced ABA, it still partially restored the synthesis of JA and SA, which was beneficial for the plant growth. The enhanced tolerance of ABA to drought is realized at the expense of plant development (Figure 1 and Figure 5), while the alleviated effects of melatonin could largely protect plant growth from drought damages (Figure 1, Figure 5 and Figure 7). Nevertheless, SGT1 and ABI5 are two crucial modules involved in this process since their mutation would prevent the effects of melatonin on drought stress (Figure 3, Figure 5 and Figure 7).

**Figure 8:**
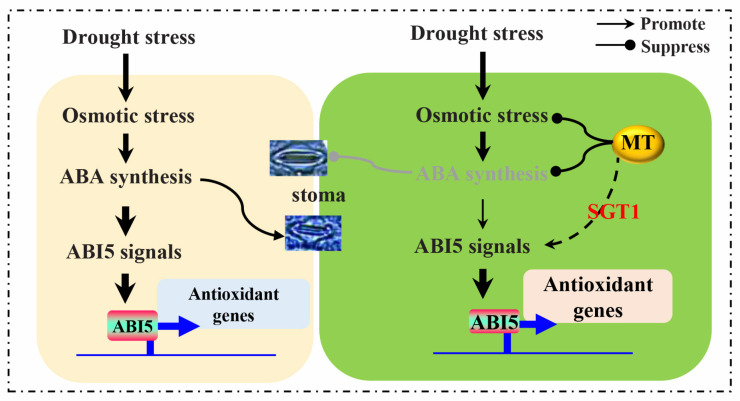
Models explaining the ameliorated effects of melatonin on seedling growth via SGT1-involved signals under drought stress in rice (*Oryza sativa* L.).

Collectively, a proposed model was given to explain the ameliorated effects of melatonin on seedlings growth via SGT1-involved signals under drought stress in rice (Figure 8). Accumulations of drought stress-induced osmolytes, i.e., SS, proline, and ABA, leads to osmosis stress and causes stoma closure, which activates ABA-mediated signals to regulate antioxidant response genes; inversely, osmosis stress intensifies the water deficiency and cause the excessive accumulation of ROS that eventually disrupt the redox homeostasis essential for cell metabolism. However, by comparison, melatonin alleviates the accumulation of osmolytes under drought stress and greatly promotes ABA-independent SGT1-involved signals to activate antioxidant genes, i.e., *OsCAT2*, which further effectively enhance the activity of the antioxidant system to scavenge ROS until reaching redox homeostasis.

## 4. Materials and Methods

### 4.1. Plant Materials and Growth Conditions

The *abi5-1* and *abi5-2* mutants were derived from Li et al. [49]. To generate *sgt1* mutants, the first exon of *OsSGT1* (*Os01g0624500*) was selected as a target (Figure S7a,b). Designing of sgRNAs and utilization of CRISPR-P program (http://cbi.hzau.edu.cn/cgi-bin/CRISPR/, accessed on 8 December 2021) were conducted following the previous methods [55], while the vector construction, as well as the *Agrobacterium-mediated* transformation of pH-*ossgt1* vector, were performed following our previously reported methods [49]. The dual mutants of *sgt-1abi5-1* and *sgt1-1abi5-2* were selected from the F_6_ generations through the cross of *sgt1-1* with *abi5-1* and *abi5-2*, respectively (Figure S7c). The cultivar *Oryza sativa* L. *japonica* was used as the wild-type (WT).

For the experimental treatments, sterilized seeds were evenly put on moisture filter papers of germinated boxes for three days, and the germinated seedlings were transferred to plastic pots containing ½MS liquid medium in a greenhouse room (12 h PAR, 500 μmol photons m^−2^ s^−1^ light/ 12 h dark; temperature settings: 28 °C, day/24 °C, night; relative humidity: 65–75%) for another 11 days growth. The ½MS liquid medium was replaced every two days. After that, part of the uniform seedlings was subjected to different levels of drought stress based on the various soil water levels at 100% field capacity (FC; as control), 80% FC, 60% FC, 40% FC, and 25% FC (as drought stress) for another seven days [56]. From the initiation of the drought treatments, different concentrations of melatonin, including 0-μmol/L, 50-μmol/L, 100-μmol/L, 200-μmol/L, and 1000-μmol/L of MT were employed to foliar spray above seedlings of 25% FC every two days. For ABA treatment, above germinated seedlings of 14 days old were transferred to ½MS liquid medium containing 10 μmol/L ABA [14] and grown under 100% FC (as ABA) or 25% FC (ABA + drought) conditions for another seven days. At the midday of the seventh day after drought treatments, the leaf samples were collected to assess the physiological parameters and gene expression levels.

### 4.2. Determination of Physiological Parameters

Relative water contents (RWC) were determined with the reported methods [57]. Survival rates were determined with the survival plant numbers to the total plant numbers within the drought stress stages. Chlorophyll (Chl.) content was determined following the previous methods [58]. Appropriately 0.3 g leaf samples were ground and resolved in 80% acetone, and absorbances of the extracts at 646 nm and 663 nm were determined to calculate the Chl. contents with a standard curve. The net photosynthetic rates (*P*_n_) were measured at the midday of day seven after drought stress using a photosynthesis system (LI-6400XT, LI-COR, Huntington Beach, CA, USA). After leaf epidermis was stained with iodine solution [1% (*w/v*) iodine and 1% (*w/v*) potassium iodide] for 30 s, stomatal morphology was observed and photographed with stereoscope (Nikon Eclipse E100, Tokyo, Japan). Stomatal conductance was measured with LI-600 stomatograph (LI-COR, Huntington Beach, CA, USA).

Soluble sugar was analyzed with the anthrone colourimetric method [59]. Briefly, 0.3 g leaf samples were homogenized with distilled water and then boiled for 30 min to collect the extracts. Subsequently, the extracts were mixed with sulfuric acid and anthrone, and the absorbances at 620 nm were detected to determine the contents of soluble sugars.

Free proline contents were detected according to the previous methods [60,61]. The homogenates collected from 0.3 g homogenized samples in 3% aqueous sulfosalicylic acid were incubated at 100 °C for 10 min. Subsequently, after centrifuging at 5000× *g* for 10 min, the supernatants were reacted with the mixture of 2.5% ninhydrin and glacial acetic acid at 100 °C for an hour and terminated in an ice bath, following a reaction with toluene. The absorbance of the chromophore phase at 520 nm was measured to determine the proline concentration.

### 4.3. Measurement of Oxidant Products

The content of hydrogen peroxide (H_2_O_2_) was determined following the methods of Velikova et al. [62] with modifications. Briefly, 0.3 g harvested leaf tissues were ground in liquid nitrogen and then immediately mixed with pre-chilled Krebs–Ringer phosphate (145 mM NaCl, 5.7 mM sodium phosphate, 4.86 mM KCl, 0.54 mM CaCl_2_, 1.22 mM MgSO_4_, 5.5 mM glucose, pH 7.35). Subsequently, the homogenate was centrifuged at 12,000× *g* for 20 min at 4°C, and then the supernatants were employed to incubate with 50 µM Amplex^®^ Red reagent (10-acetyl-3,7-dihydrophenoxazine, ThermoFisher, Shanghai, China) and 0.1 U/mL horseradish peroxidase (HRP, ThermoFisher, Shanghai, China) at 37 °C for 10 min away from light. The fluorescence was measured at 530 nm and 590 nm using a VersaFluor fluorometer (Bio-Rad, Hercules, CA, USA) and calculated based on a standard curve constructed using known concentrations of H_2_O_2_.

MDA was detected with the previous methods [63]. Briefly, 0.3 g of homogenized samples were mixed with 10% trichloroacetic acid (*v/v*) and was centrifuged at 12,000× *g* for 20 min. The supernatant was added with thiobarbituric acid under a water bath of 95 °C for 30 min and centrifuged at 12,000× *g* for 30 min. The supernatants were collected to measure absorbance at 450, 532, and 600 nm, respectively.

The superoxide (O_2_^−^) production rates were measured using the reported methods [64]. Briefly, 0.3 g of homogenized samples were mixed with 65 mM phosphate-buffered saline (PBS, pH 7.8). After centrifuging at 10,000× *g* for 30 min at 4 °C, the supernatant was transferred to mix with 10 mM hydrochloride and subsequently added with 58 mM sulfanilamide and 7 mM a-naphthylamine and incubated at 25 °C for 20 min. Finally, chloroform was added, and the absorbance at 530 nm was measured.

### 4.4. Activity Assays of Antioxidant System

The activity of superoxide dismutase (SOD, EC 1.15.1.1) and catalase (CAT, EC 1.11.1.6) was determined with the previous methods [49,65], while activities of ascorbate peroxidase (APX, EC 1.11.1.11) and glutathione reductase (GR, EC 1.6.4.2) were measured according to the previous methods [66]. Briefly, the homogenizing power of 0.3 g samples was suspended with 50 mM pre-cold phosphate buffer (containing 0.2 mM EDTA, 2% polyvinylpyrrolidone (*w/v*), pH 7.8). After centrifuging at 4 °C for 10 min (12,000× *g*), the supernatants were collected to determine the activity of SOD, CAT, APX and GR.

Extractions of glutathione (GSH) and ascorbic acid (ASA) were conducted with 0.3 g leaf samples following the previous methods [21]. In brief, 0.3 g of fresh leaf tissues were ground to a powder in liquid nitrogen and then mixed with a precooled solution containing 0.1 mol/L phosphate buffer (pH 7.33). The supernatants were collected after centrifugation (10 min at 3000× *g*) and used for the determination of the GSH and ASA contents by using the enzyme-linked immunosorbent assay (ELISA) method with commercial kits (Nanjing Jiancheng Bio. Institute, Nanjing, China).

### 4.5. Measurement of ABA, SA and JA Contents

Quantification of ABA, SA and JA were analyzed according to the previous methods [19,67,68] with few modifications. Briefly, 0.3 g samples were ground and incubated with pre-cold extraction solution (containing methanol/water/formic, 16:3:1, *v*/*v*/*v*, 1 mM butylated hydroxytoluene). After centrifuging at 10,000× *g* for 15 min at 4 °C, the supernatant was filtered through a Sep-Pak C18 cartridge (Waters, Milford, MA, USA). Thereafter, filters were collected to determine the contents of ABA, JA and SA by using LC-MS analysis (Aligent 6460 triple Quad LC/MS platform, Denver, CO, USA).

### 4.6. Gene Expression Analysis

The total RNA was extracted with 0.3 g leaf samples with the Qiagen Spin Plant RNA Kit (Tiangen, Beijing, China) according to the manufacturer’s instructions. Quantitative Realtime PCR (qRT-PCR) analysis was performed using the 2^−ΔΔCt^ method (Livak and Schmittgen, 2001) [69], and the expression level was calculated relative to rice *UBQ5* gene according to the previous methods [70]. Genes were identified through homology search with the reported *Arabidopsis* genes by using the Rice Annotation Project (RAP) Database (https://rapdb.dna.affrc.go.jp/, accessed on 8 December 2021): *Abscisic acid insensitive 5* (*OsABI5*, Os01g0859300); *Suppressor of the G2 allele of skp1* (*OsSGT1*, Os01g0624500); *Basic helix-Loop-helix protein 6* (*OsbHLH6*, Os10g0575000); *Myelocytomatosis oncogene transcription factor 2* (*OsMYC2*, Os10g0575000); *Superoxide dismutase A1* (*OsSODA1,* Os05g0323900); *Peroxisomal ascorbate peroxidase 4* (*OsAPX4,* Os08g0549100); *Catalase 2* (*OsCAT2,* Os02g0115700); *Glutathione reductase 2* (*OsGR2,* Os02g0813500). These genes were subjected to qRT-PCR analysis using gene-specific primers (Table S1).

### 4.7. Statistical Analysis

Values were determined with six biological replicates and showed as means ± standard deviations. The one-way ANOVA tests were used for data comparisons from different groups, followed by Tukey’s Multiple Comparison Test (*p* < 0.05).

## 5. Conclusions

We here demonstrated that melatonin promoted SGT1-mediated signals to ameliorate drought stress, probably via ABI5-mediated expression of antioxidant genes, e.g., *OsCAT2*, which could promote the ROS scavenging until reaching ROS homeostasis.

## Data Availability

All data generated or analyzed during this study are included in this published article.

## Supplementary Materials

The following are available online at https://www.mdpi.com/article/10.3390/ijms23020599/s1.
