# Supplementary material for: Melatonin Promotes SGT1-Involved Signals to Ameliorate Drought Stress Adaption in Rice
Source: Int J Mol Sci. 2022 Jan 6;23(2):599. doi: 10.3390/ijms23020599 (PMC8775989; doi:10.3390/ijms23020599)
Supplement: Supplementary file 1 [file ijms-23-00599-s001.zip › ijms-1524522-supplementary.pdf]

Figure S1

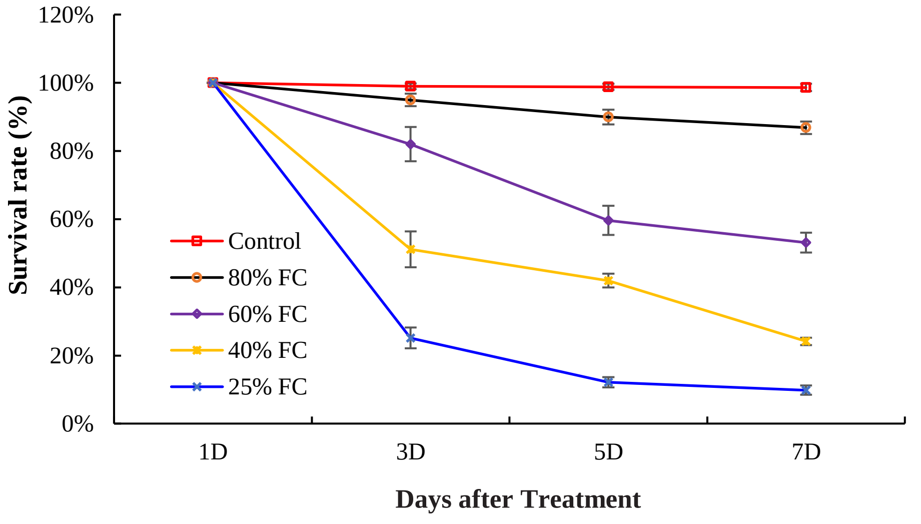

Figure S1 Effects of various levels of drought stress on the survival rates. Applications of different concentrations of field capacity (100%::Control, 80%-, 60%-, 40%- and 25%-FC) were employed to treat 14 days old seedlings for another 7 days.

Figure S2

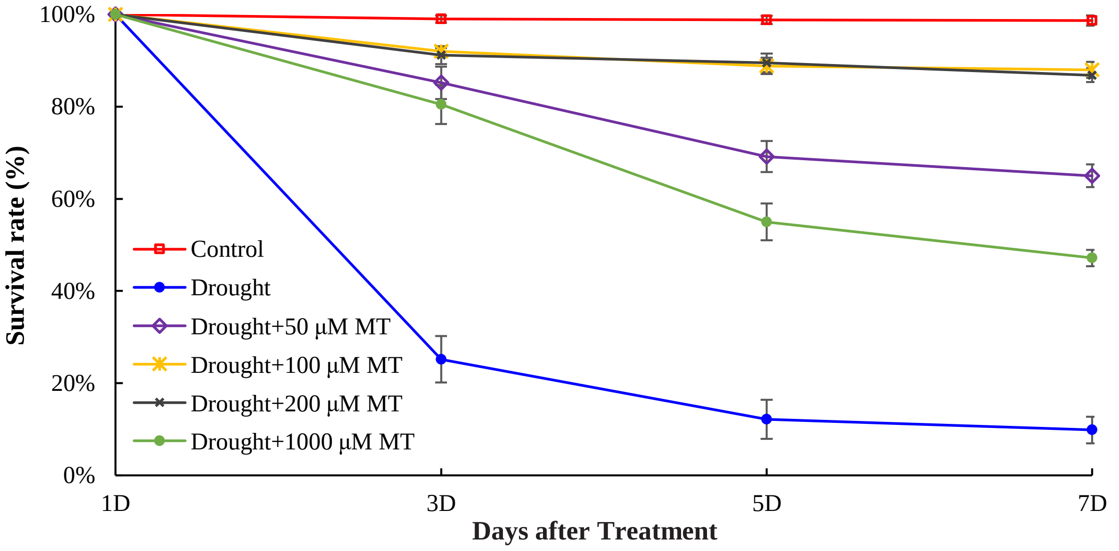

Figure S2 Effects of different concentrations of melatonin on the survival rates under severe drought stress.

Applications of different concentrations of exogenous melatonin (MT; 0, 50, 100, 200 and 1000 µM) were employed to pretreat 14 days old seedlings for another 7 days under drought stress (25% field capacity; FC) .

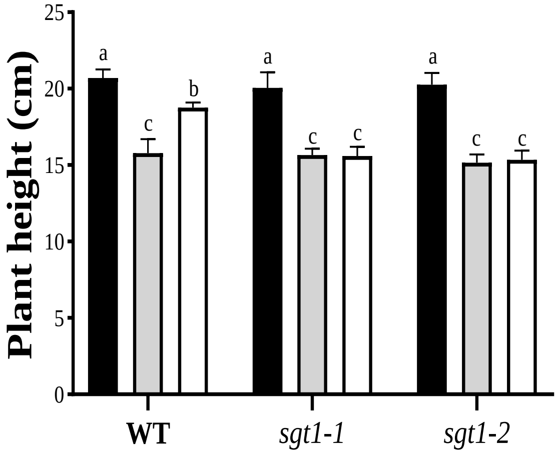

Figure S3 Effects of melatonin treatments on plant height in *sgt1* mutant under drought stress.

Figure S4

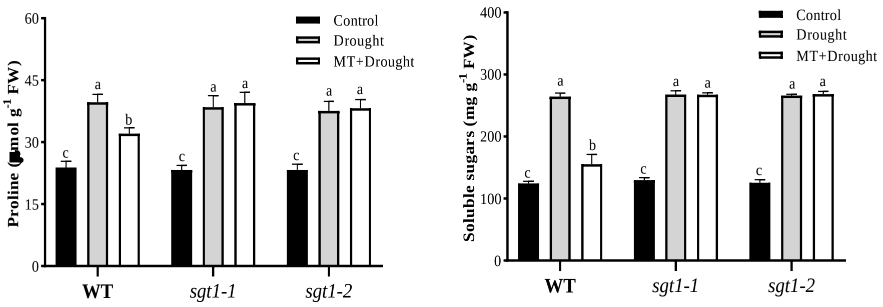

Figure S4 Effects of melatonin treatments on the contents of proline and soluble sugars in *sgt1* mutant under drought stress.

Figure S5

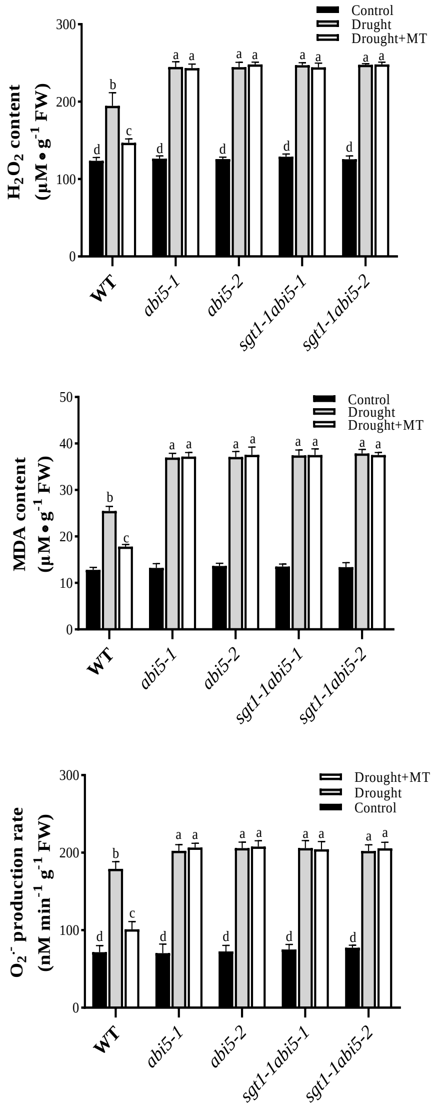

Figure S5 Effects of melatonin treatments on the oxidative damages of *abi5* and *abi5sgt1* under drought stress.

Contents of hydrogen peroxide (H_2O_2), malondialdehyde (MDA), and superoxide ($O_2^{\cdot -}$) production rates, were examined in the leaves of wild-type , *abi5*, and *sgt1abi5* mutant seedlings pretreated with melatonin (MT; 100 μM) under drought stress.

Figure S6

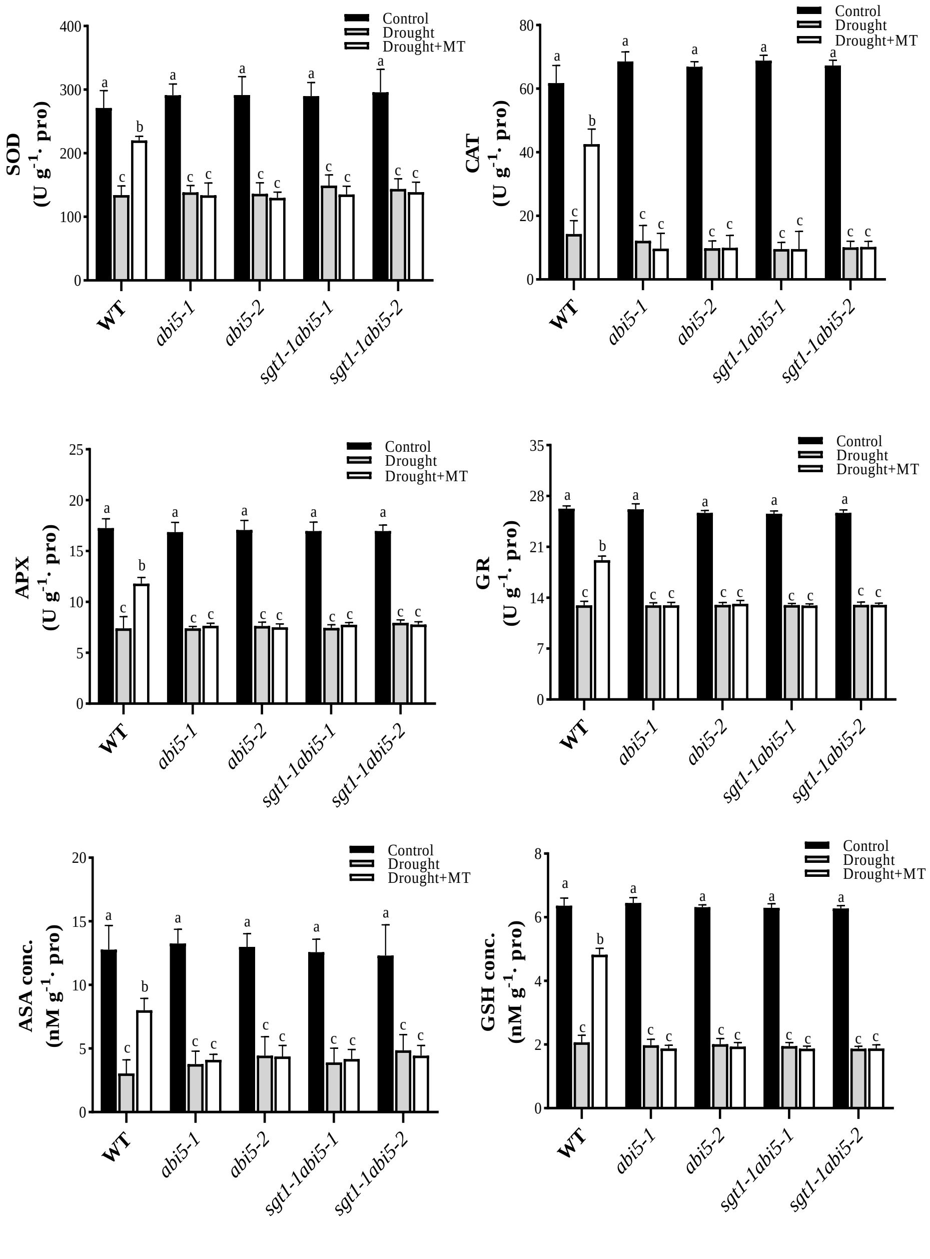

Figure S6 Effects of melatonin treatments on the antioxidant activity of *abi5* and *abi5sgt1* under drought stress.

Activities of superoxide dismutase (SOD), peroxidase (POD), ascorbate (APX), catalase (CAT), glutathione reductase (GR), and the glutathione (GSH) contents, were examined in the leaves of wild-type, *abi5*, and *sgt1abi5* mutant seedlings pretreated with melatonin (MT; 100 μM) under drought stress.

Figure S7

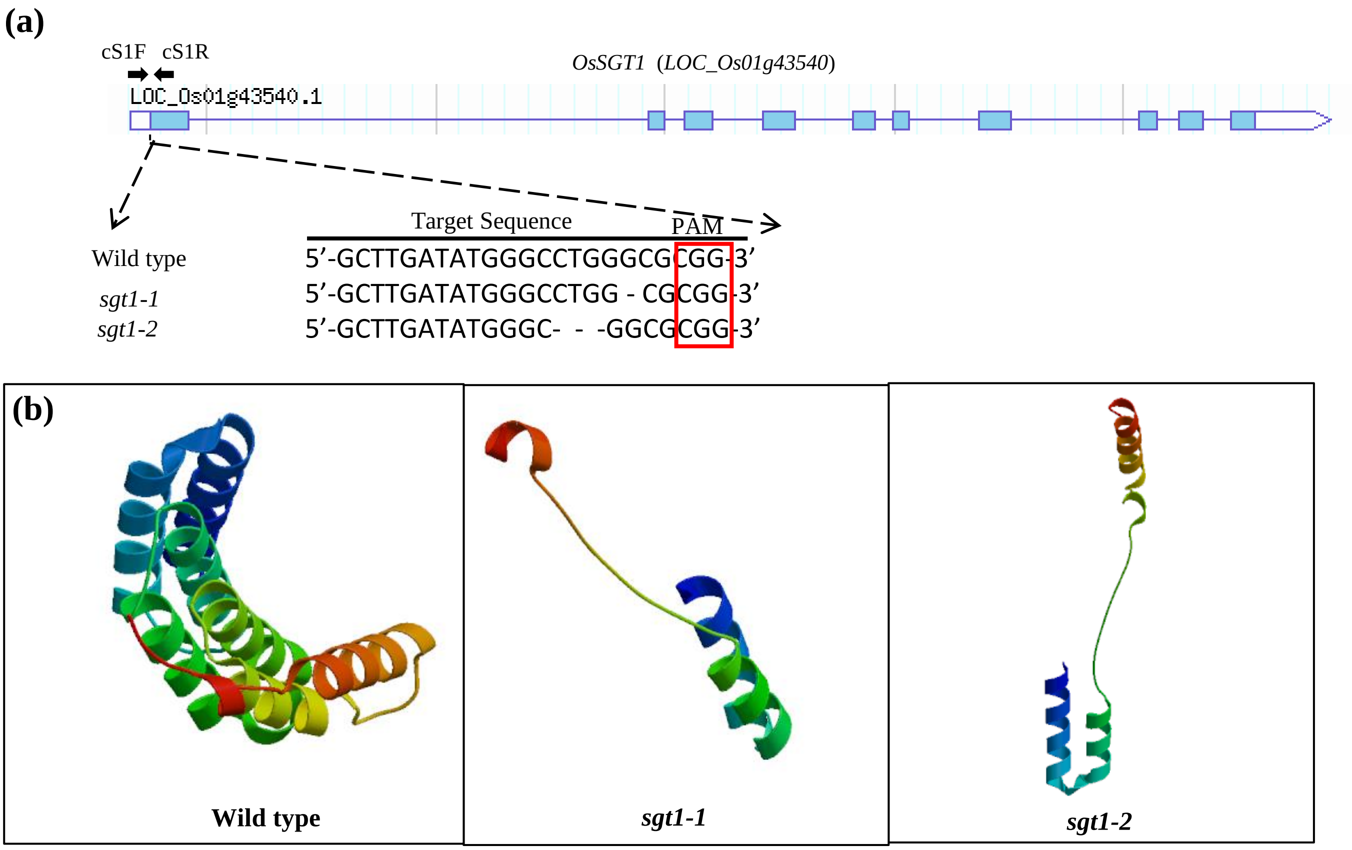

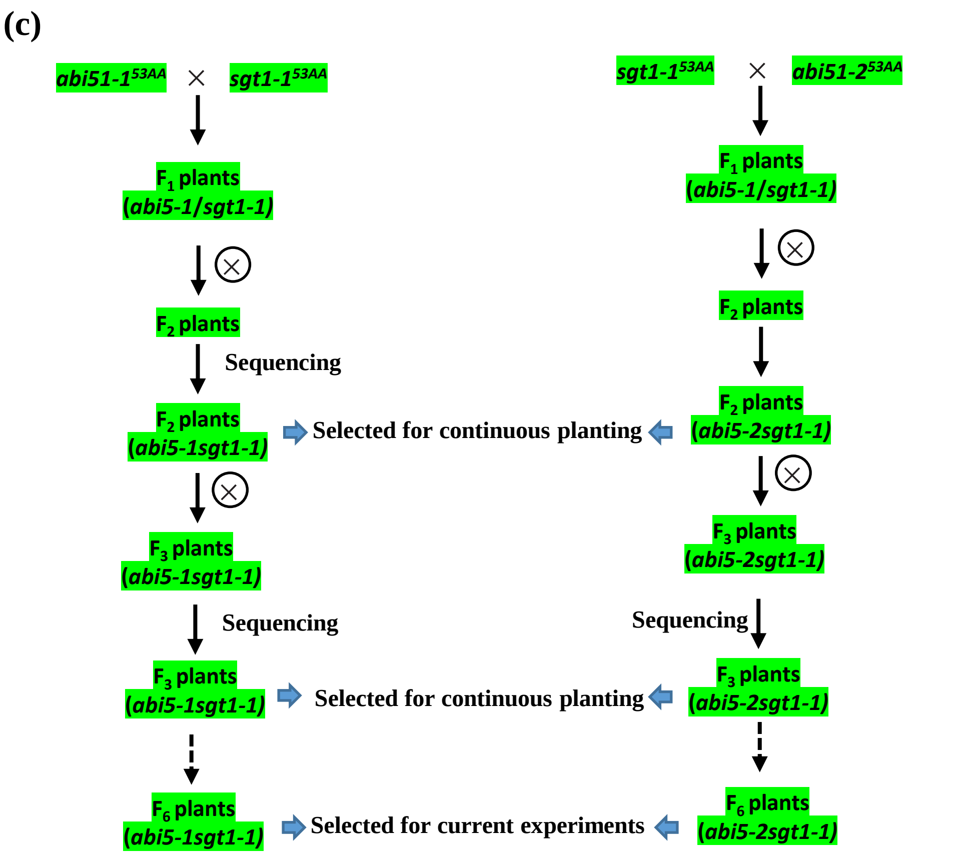

Figure S7 Schematic diagram of *OsSGT1* and sgRNA target site for CRISPR/Cas9-mediated mutagenesis.
(a) Exons, introns and UTRs are indicated by solid boxes, lines and blank boxes, respectively. cS1F and cS1R are primers for genotyping mutation, and its position are indicated by arrowheads. Mutation identified within the target site of *OsABI5* generated through CRISPR/Cas9-mediated genome editing in rice. The PAM sequences (NGG) are boxed and the 20-nt target sequences are underlined. Mutations are shown in '–' (for deletion). (b) The 3-dimensional structures of OsABI5 were analyzed on SWISS-MODEL (https://www.swissmodel.expasy.org/). (c) Breeding information of *sgt1-1abi5-1 and sgt1-1abi5-2* used in the present study.

Table S1 Primers used in this study

Name	Forward Primer Sequence (5'→3')	Reverse Primer Sequence (5'→3')
UBQ5	CCGACTACAACATCCAGAAGGAG	AACAGGAGCCTACGCCTAAGC
L64000 pHUC411-sg2.0	GGCATCTCACCTAGTGTTACCTGT	AAACACAGGTAACACTAGGTGAGA
L43540 pHUC411-sg2.0	GGCAGCTTGATATGGGCCTGGGCG	AAACCGCCCAGGCCCATATCAAGC
ABI5	GATCATTTTCCTTGCCGCTAC	CAAGTGTCATCTCACCTAGTGT
MYC2	TGGAGGCCATGATC	CGTTGAGCTGGTC
bHLH6	TAGTGTCCAAATGAAGC	CTCATGGAGCTCAACGG
SODA1	AAACAACTGCTAACCA	TCCAGTTCATCACCTT
APX4	TGCATCTACGAAATCTG	CTTCTTAGAAGCCTC
GR2	TTATCCAGGGTATGGC	TGGCTTGGATGATG
CAT2	CCTATGCTGATACCCAAAG	CACACTGCGACCAGTAGGA
SGT1	TACAGGCACGACTTCT	GTACGGCTCCTCTCCA
cN2	ATCATTTTCCTTGCCGCTACCG	ATCCAGGACTCACGACAACCAT
cS1	AAGTCTTCGTCTGGTCTTCCCC	TCTCCTCCTATCAGCAAGTCAA
